# The Alzheimer's Disease Neuroimaging Initiative‐4 (ADNI‐4) Engagement Core: A culturally informed, community‐engaged research (CI‐CER) model to advance brain health equity

**DOI:** 10.1002/alz.14242

**Published:** 2024-10-23

**Authors:** Mónica Rivera Mindt, Alyssa Arentoft, Amanda T. Calcetas, Vanessa A. Guzman, Hannatu Amaza, Adeyinka Ajayi, Miriam T. Ashford, Omobolanle Ayo, Lisa L. Barnes, Alicia Camuy, Catherine Conti, Adam Diaz, Bashir Easter, David J. Gonzalez, Yolanda Graham Dotson, Isabella Hoang, Kaori Kubo Germano, Gladys E. Maestre, Fabiola Magaña, Oanh L. Meyer, Melanie J. Miller, Rachel Nosheny, Van M. Ta Park, Shaniya Parkins, Lisa Renier Thomas, Joe Strong, Sandra Talavera, Steven P. Verney, Trinity Weisensel, Michael W. Weiner, Ozioma C. Okonkwo, the Alzheimer's Disease Neuroimaging Initiative

**Affiliations:** ^1^ Department of Psychology Fordham University Bronx New York USA; ^2^ Department of Latin American and Latino Studies Institute and Department of African and African American Studies Fordham University Bronx New York USA; ^3^ Department of Neurology Icahn School of Medicine at Mount Sinai New York New York USA; ^4^ Department of Psychology California State University, Northridge Northridge California USA; ^5^ Department of Medicine, Wisconsin Alzheimer's Disease Research Center School of Medicine and Public Health University of Wisconsin‐Madison Madison Wisconsin USA; ^6^ Northern California Institute for Research and Education (NCIRE) San Francisco California USA; ^7^ VA Advanced Imaging Research Center Veterans Affairs Medical Center San Francisco California USA; ^8^ Department of Neurological Sciences Rush University Medical Center Chicago Illinois USA; ^9^ Community Science Partnership Board (CSPB) Alzheimer's Disease Neuroimaging Initiative New York New York USA; ^10^ Melanin Minded LLC Milwaukee Wisconsin USA; ^11^ Indiana Alzheimer's Disease Research Center School of Medicine Indiana University Indianapolis Indiana USA; ^12^ Department of Neuroscience University of Texas Rio Grande Valley School of Medicine Rancho Viejo Texas USA; ^13^ Department of Neurology School of Medicine University of California Davis Sacramento California USA; ^14^ Department of Psychiatry and Behavioral Sciences University of California San Francisco San Francisco California USA; ^15^ Department of Radiology and Biomedical Imaging University of California San Francisco San Francisco California USA; ^16^ Department of Community Health Systems School of Nursing University of California San Francisco San Francisco California USA; ^17^ Asian American Research Center on Health (ARCH) University of California San Francisco San Francisco California USA; ^18^ Department of Psychology University of New Mexico Albuquerque New Mexico USA

**Keywords:** Alzheimer's disease, cognitive aging, community‐engaged research, cultural humility, dementia, diversity, external validity, generalizability, health disparities, health equity, inclusion science, inclusive participation, underrepresented populations

## Abstract

**INTRODUCTION:**

The Alzheimer's Disease Neuroimaging Initiative‐4 (ADNI‐4) Engagement Core was launched to advance Alzheimer's disease (AD) and AD‐related dementia (ADRD) health equity research in underrepresented populations (URPs). We describe our evidence‐based, scalable culturally informed, community‐engaged research (CI‐CER) model and demonstrate its preliminary success in increasing URP enrollment.

**METHODS:**

URPs include ethnoculturally minoritized, lower education (≤ 12 years), and rural populations. The CI‐CER model includes: (1) culturally informed methodology (e.g., less restrictive inclusion/exclusion criteria, sociocultural measures, financial compensation, results disclosure, Spanish Language Capacity Workgroup) and (2) inclusive engagement methods (e.g., the Engagement Core team; Hub Sites; Community–Science Partnership Board).

**RESULTS:**

As of April 2024, 60% of ADNI‐4 new in‐clinic enrollees were from ethnoculturally or educationally URPs. This exceeds ADNI‐4's ≥ 50% URP representation goal for new enrollees but may not represent final enrollment.

**DISCUSSION:**

Findings show a CI‐CER model increases URP enrollment in AD/ADRD clinical research and has important implications for clinical trials to advance health equity.

**Highlights:**

- The Alzheimer's Disease Neuroimaging Initiative‐4 (ADNI‐4) uses a culturally informed, community‐engaged research (CI‐CER) approach.
- The CI‐CER approach is scalable and sustainable for broad, multisite implementation.
- ADNI‐4 is currently exceeding its inclusion goals for underrepresented populations.

## 1 BACKGROUND

Alzheimer's Disease (AD) and AD‐related Dementia (ADRD) differentially impact underrepresented populations (URPs), particularly ethnoculturally minoritized groups (e.g., American Indian/Alaska Native, Asian/Asian American, Black, Hispanic/Latina/o/e/x (herein Latinx), Native Hawaiian/Other Pacific Islander). In the United States, URPs experience higher prevalence and incidence of AD/ADRD and worse long‐term cognitive and functional outcomes.^1^, ^2^, ^3^, ^4^, ^5^, ^6^, ^7^ Black and Latinx adults are up to twice as likely to develop AD/ADRD compared to non‐Latinx White (NLW) adults.^8^ URPs bear a disparate burden of sociocultural determinants of health that increase dementia risk, but remain poorly represented in AD/ADRD research.^9^, ^10^, ^11^, ^12^, ^13^, ^14^, ^15^, ^16^, ^17^, ^18^

URPs were profoundly under‐included in AD research from nine major cohort studies, including the Alzheimer's Disease Neuroimaging Initiative (ADNI), suggesting sampling bias^9^ and illustrating the disconnect between AD/ADRD burden versus research inclusion. Over the past 20 years, ADNI's cohorts have been > 80% NLW.^19^, ^20^ Despite National Institutes of Health recommendations to prioritize AD/ADRD inequities,^21^ few studies focus on URPs,^1^, ^19^, ^22^, ^23^, ^24^, ^25^, ^26^, ^27^, ^28^, ^29^, ^30^, ^31^, ^32^, ^33^ primarily enrolling NLW adults instead through convenience sampling.^34^, ^35^ Further, studies have historically failed to retain URPs, who are less likely to engage in genetic and biomarker research and participate in longitudinal follow‐ups.^36^, ^37^, ^38^, ^39^, ^40^, ^41^, ^42^ Both observational and interventional studies have struggled to effectively include and retain URPs.^24^, ^34^, ^41^, ^43^, ^44^, ^45^, ^46^, ^47^, ^48^, ^49^, ^50^, ^51^, ^52^

The Culturally Informed, Community‐Engaged Research (CI‐CER) model^30^, ^31^ is an evidence‐based approach for more successful URP recruitment and retention (herein referred to as inclusion and engagement), based on Community‐Based Participatory Research (CBPR) principles, which has been scaled for large, multisite, and/or decentralized studies. Research suggests that CI‐CER—and related approaches across the continuum of participatory methods—significantly increase URP inclusion and engagement.^53^, ^54^ Table 1 illustrates the epistemological and ontological differences between traditional, non‐participatory clinical research compared to our CI‐CER model. This approach emphasizes sustained collaboration and involvement with communities throughout the research process, from conception to dissemination.^30^, ^53^, ^55^, ^56^ Inclusion and engagement strategies guided by CI‐CER principles establish genuine, fair partnerships and recognize communities’ strengths, assets, and identities. CI‐CER emphasizes the training and engagement of culturally competent research teams with the requisite cultural and linguistic skills to work with specific URPs and maintain consistent engagement over time, which includes sharing research findings and knowledge gained with community members.^57^, ^58^ This co‐learning process benefits a greater proportion of minoritized individuals who have been historically excluded from research. Overall, CI‐CER requires long‐term, reciprocal, authentic relationships between researchers and communities.

**RESEARCH IN CONTEXT**

1. **Systematic review**: Study authors reviewed relevant literature through academic search engines and databases (e.g., Google Scholar, PubMed). The authors detail the methods implemented in Alzheimer's Disease Neuroimaging Initiative‐4 (ADNI‐4)’s culturally informed, community‐engaged research (CI‐CER) approach, including culturally informed methodology and inclusive participation and engagement methods
2. **Interpretation**: ADNI‐4's CI‐CER approach, thus far, has increased the inclusion and engagement of underrepresented populations.
3. **Future directions**: ADNI‐4's CI‐CER approach provides a blueprint for other larger‐scale multisite studies and Alzheimer's disease clinical trials to implement equitable inclusion and engagement strategies at scale.

**TABLE 1:** Epistemological and ontological prism of traditional clinical research compared to culturally informed, community‐engaged research (CI‐CER).

Epistemological and ontological prism		
	Traditional clinical research	CI‐CER
Who		
Researcher's role	“Conquistador”	Partner
Action orientation	Get	Give
Power/leadership	Retained by researchers	Shared with community
What		
Study/team structure	More vertical/patriarchal	More lateral/matriarchal
Goal orientation	Academically driven	Community informed
Knowledge	Retained by researchers	Shared with community
Where		
Research site(s)	Ivory towers (e.g., academic medical centers)	Community (in‐person or digital)
When		
Time orientation	Go fast and time‐limited	Go slower and sustained involvement

Successful CI‐CER involves a shift in investments of time and funding toward this work, a culturally competent and culturally humble scientific workforce, and CI‐CER and health equity experts to lead such efforts.^31^ Despite empirical support, CI‐CER methods have not been widely adopted or scaled for multisite implementation in AD/ADRD research. However, in 2020, ADNI‐3 launched the Diversity Task Force (DVTF; co‐led by Drs. Okonkwo and Rivera Mindt), a 2‐year pilot study to test whether a comprehensive CI‐CER approach deployed at 13 sites would increase URP inclusion;^30^ see Weiner et al.^59^ The DVTF was a success; at the 13 DVTF sites, 94% of newly enrolled participants during the pilot period were from a URP background. Consequently, ADNI‐4 launched the new Engagement Core, with scaled and expanded CI‐CER methods. The aims of the Engagement Core are to:

1. Ensure that ≥ 50% of all newly enrolled ADNI‐4 participants are from URPs;
2. Cultivate AD/ADRD workforce capacity through:
  - Ongoing training of all ADNI‐4 leadership and team members in cultural competence and humility, and CI‐CER methods,
  - Establishing the Health Equity Scholars Program (HESP; see Amaza et al.),^60^ a new, national training program in AD/ADRD and brain health equity; and
3. Advance brain health equity research in ADNI‐4 using a CI‐CER model.

This paper aims to:

1. Describe and operationalize ADNI‐4's scalable, evidence‐based CI‐CER approach, which includes:
  - Culturally informed methodology (e.g., less restrictive inclusion/exclusion criteria, sociocultural measures, financial compensation, results disclosure, Spanish Language Capacity Workgroup); and
  - Inclusive participation and engagement methods (i.e., the Engagement Core team; Hub Sites; Community Science Partnership Board (CSPB); and CI‐CER–based digital engagement), and
2. Evaluate ADNI‐4's preliminary in‐clinic inclusion data for new enrollees from URPs.

## 2 METHODS

### 2.1 Participants

ADNI is a longitudinal, multisite project spanning four prior phases (ADNI‐1, ADNI‐GO, ADNI‐2, and ADNI‐3) over the past 20 years. ADNI's primary goal is to validate AD biomarkers for clinical trials and it continues to investigate predictors of neurodegeneration and cognitive decline, particularly AD/ADRD progression, and the methods used and participants enrolled in ADNI have been described in detail elsewhere (adni.loni.usc.edu; see Weiner et al. in this special issue for further details). Therefore, this paper will focus only on methods specifically relevant or unique to ADNI‐4, and include ADNI‐4 data obtained between July 2023 (i.e., first participant enrollment in ADNI‐4) and April 11, 2024.

ADNI‐4 aims to have up to 1500 participants join the in‐clinic study. Approximately 500 to 750 new participants will be enrolled for in‐clinic evaluations and ≈ 500 to 750 additional rollover participants from ADNI‐3 will be followed longitudinally into ADNI‐4. ADNI‐4 also includes new remote cohorts to help bolster recruitment and ultimately referral of new participants to clinical sites. This will include a remote digital cohort that follows participants longitudinally, and will screen at least 20,000 individuals through a brief online screener and subsequently enroll ≈ 4000 into blood biomarker screening (i.e., the remote blood cohort; see Miller et al.).^61^ All sites are asked to contact ADNI‐3 participants to encourage them to continue in ADNI‐4 and are provided with materials to share with participants (e.g., a certificate and thank you letter for ADNI‐3 participation, informational flyer describing ADNI‐4 and the continuation [i.e., rollover] process). The scientific value of rollover participation for the collection of longitudinal data is emphasized. For ADNI‐4, a new flyer was developed specifically for rollovers from URP backgrounds (as well as potential new participants) using diverse imagery and jargon‐free language.

The primary aim of the Engagement Core is to ensure that at least 50% of newly enrolled in‐clinic participants are from URPs. For ADNI‐4, URP status is operationalized as persons who self‐report being from at least one of the following backgrounds:

a. Ethnoculturally minoritized group, including: American Indian or Alaska Native, Asian/Asian American, Black or African American, Latinx, and/or Native Hawaiian or other Pacific Islander;
b. Lower educational background (i.e., ≤12 years of education); or
c. Rural geographic location based on rural–urban commuting area (RUCA) score ≥ 4.^62^

#### 2.1.1 Inclusion criteria

ADNI‐4's inclusion and exclusion criteria are less restrictive than previous ADNI phases, in which URPs were disproportionately excluded, in part due to increased rates of comorbidities.^20^, ^27^ Changes in inclusion and exclusion criteria were piloted during the ADNI‐3 DVTF efforts, with the support of ADNI‐3 leadership, which informed the criteria detailed below; please see ADNI‐4 in‐clinic protocol for further details. All newly enrolled participants in the in‐clinic cohort, regardless of diagnostic category, must meet the following criteria: between the ages of 55 and 90 years old; Geriatric Depression Scale score < 10; sufficient visual and auditory acuity for neuropsychological testing; literate and fluent in English or Spanish; and not pregnant, lactating, or of childbearing potential. Participants must be willing and able to participate in a longitudinal study and agree to study procedures, including blood and biomarker testing (e.g., genome‐wide association studies, apolipoprotein E, SNA, RNA), cognitive testing, magnetic resonance imaging (MRI) and positron emission tomography (PET) scans. In addition, all newly enrolled in‐clinic cohort participants must meet specific inclusion criteria by diagnostic category, as outlined in Table 2. Rollover participants (i.e., participants enrolled in prior ADNI phases who are continuing in ADNI‐4) must have been enrolled in ADNI for at least 1 year. With approval from ADNI leadership, criteria may be relaxed for those who have lived for ≥ 8 years in a low socioeconomic status neighborhood. Additionally, ADNI‐4 in‐clinic participants will identify a study partner with whom they are in frequent contact (i.e., at least 2 hours of contact per week, a reduced time requirement from prior phases of ADNI) and who may accompany them to study visits or provide information remotely. Lastly and notably, lumbar puncture (LP) is no longer an inclusion criterion, although LP completion remains highly encouraged.

**TABLE 2:** Additional inclusion and exclusion criteria for the ADNI‐4 in‐clinic cohort by diagnostic category.

Cognitively normal	Mild cognitive impairment	Dementia
**Inclusion criteria**		
Participant may or may not have a significant subjective memory concern as reported by participant, study partner, or clinician.	Participant must have a subjective memory concern as reported by participant, study partner, or clinician.	Same as MCI
Normal memory function documented by scoring above demographically adjusted cutoffs on the Logical Memory II subscale (Delayed Paragraph Recall, Paragraph A only) from the Wechsler Memory Scale—Revised (the maximum score is 25): ≥ 9 for ≥ 16 years of education. ≥ 5 for 8–15 years of education. ≥ 3 for 0–7 years of education. Note: cut‐offs may be modified over time as the field evolves in this area.	Abnormal memory function documented by scoring within the demographically adjusted ranges on the Logical Memory II subscale (Delayed paragraph recall, Paragraph A only) from the Wechsler Memory Scale—Revised (the maximum score is 25): ≤ 11 for ≥ 16 years of education. ≤ 9 for 8–15 years of education. ≤ 6 for 0–7 years of education. d. Note: cut‐offs may be modified over time as the field evolves in this area.	Same as MCI
MMSE score between 24 and 30 (inclusive; exceptions may be made for participants with < 8 years of education at the discretion of the Project Director and/or Clinical Core).	Same as CN	MMSE score between 20 and 28 (inclusive; exceptions may be made for participants with < 8 years of education at the discretion of the Project Director and/or Clinical Core).
Clinical Dementia Rating = 0. Memory box score must be 0.	Clinical Dementia Rating = 0.5. Memory box score must be at least 0.5.	Clinical Dementia Rating = 0.5 or 1.0.
CN, based on an absence of significant impairment in cognitive functions or activities of daily living.	General cognition and functional performance sufficiently preserved such that a diagnosis of dementia cannot be made by the site physician at the time of the screening visit.	Meets the National Institute on Aging/Alzheimer's Association Diagnostic Guidelines for Dementia (2011).
Stability of permitted medications for 4 weeks. In particular, participants may take: Stable doses of antidepressants lacking significant anticholinergic side effects (if they are not currently depressed and do not have a history of major depression within the past 1 year). Estrogen replacement therapy. Gingko biloba, but taking it is discouraged. Washout from psychoactive medication (e.g., excluded antidepressants, neuroleptics, chronic anxiolytics or sedative hypnotics) for at least 4 weeks prior to screening.	Same as CN + e. Cholinesterase inhibitors and memantine are allowable if stable for 12 weeks prior to screen. f. Aducanumab and any other approved treatments for the neurobiology of AD if stable for 24 weeks prior to screen. Sites encouraged to collect baseline visit data prior to when participants start approved anti‐amyloid treatments.	Same as MCI
**Exclusion criteria**		
Any significant neurologic disease, such as Parkinson's disease, vascular cognitive impairment/dementia, Huntington's disease, normal pressure hydrocephalus, brain tumor, progressive supranuclear palsy, seizure disorder, subdural hematoma, multiple sclerosis, or history of significant head trauma followed by persistent neurologic defaults or known structural brain abnormalities.	Any significant neurologic disease other than suspected AD, such as Parkinson's disease (Parkinsonian symptoms complicating MCI/AD are acceptable), vascular cognitive impairment dementia (multiple lacunes ≤ 1.5 cm and/or extensive white matter changes are acceptable), Huntington's disease, normal pressure hydrocephalus, brain tumor (clinically insignificant meningioma acceptable), progressive supranuclear palsy, seizure disorder, subdural hematoma, multiple sclerosis, or history of significant head trauma followed by persistent neurologic defaults or known structural	Same as MCI.

Abbreviations: AD, Alzheimer's disease; CN, cognitively normal; MCI, mild cognitive impairment; MMSE, Mini‐Mental State Examination.

#### 2.1.2 Exclusion criteria

New in‐clinic participants are excluded for any of the following: screening/baseline MRI shows infection or clinically significant lesions (excluding cortical strokes that do not distort anatomy, lacunar infarction, and white matter disease); screening/baseline MRI shows large structural abnormalities incompatible with imaging analytics (e.g., large infarcts, large arachnoid cysts, large areas of encephalomalacia); unable or unwilling to undergo MRI (e.g., claustrophobia, implanted metal device incompatible with site MRI); a major depressive or bipolar episode within the past year; psychotic or related symptoms (e.g., agitation), sufficient to interfere with protocol compliance, within 3 months; taking medication for obsessive‐compulsive disorder or attention deficit hyperactivity disorder; history of schizophrenia; history of alcohol or substance use disorder within the past 2 years; unstable medical condition or major systemic illness sufficient to interfere with protocol compliance (including clinically significant abnormalities in B12 or thyroid function); residence in a skilled nursing facility; taking investigational medications for longer than 1 month prior to study entry (or five medication half‐lives, if longer) or at any time during the duration of their participation; participating in any other studies involving neuropsychological testing more than once per year. Further, participants may be excluded if they are taking certain psychoactive or other medications (e.g., specific neuroleptic, hypnotic), at the evaluating clinician's discretion. Criteria may be relaxed for those who have lived for ≥ 8 years in a low socioeconomic status neighborhood. Of note, psychiatric diagnoses are based on Diagnostic and Statistical Manual of Mental Disorders, Fifth Edition criteria.

### 2.2 Procedures

The following procedures and methods will focus on ADNI‐4's Engagement Core. All other standard ADNI‐4 procedures are described elsewhere.^20^

**FIGURE 1:**
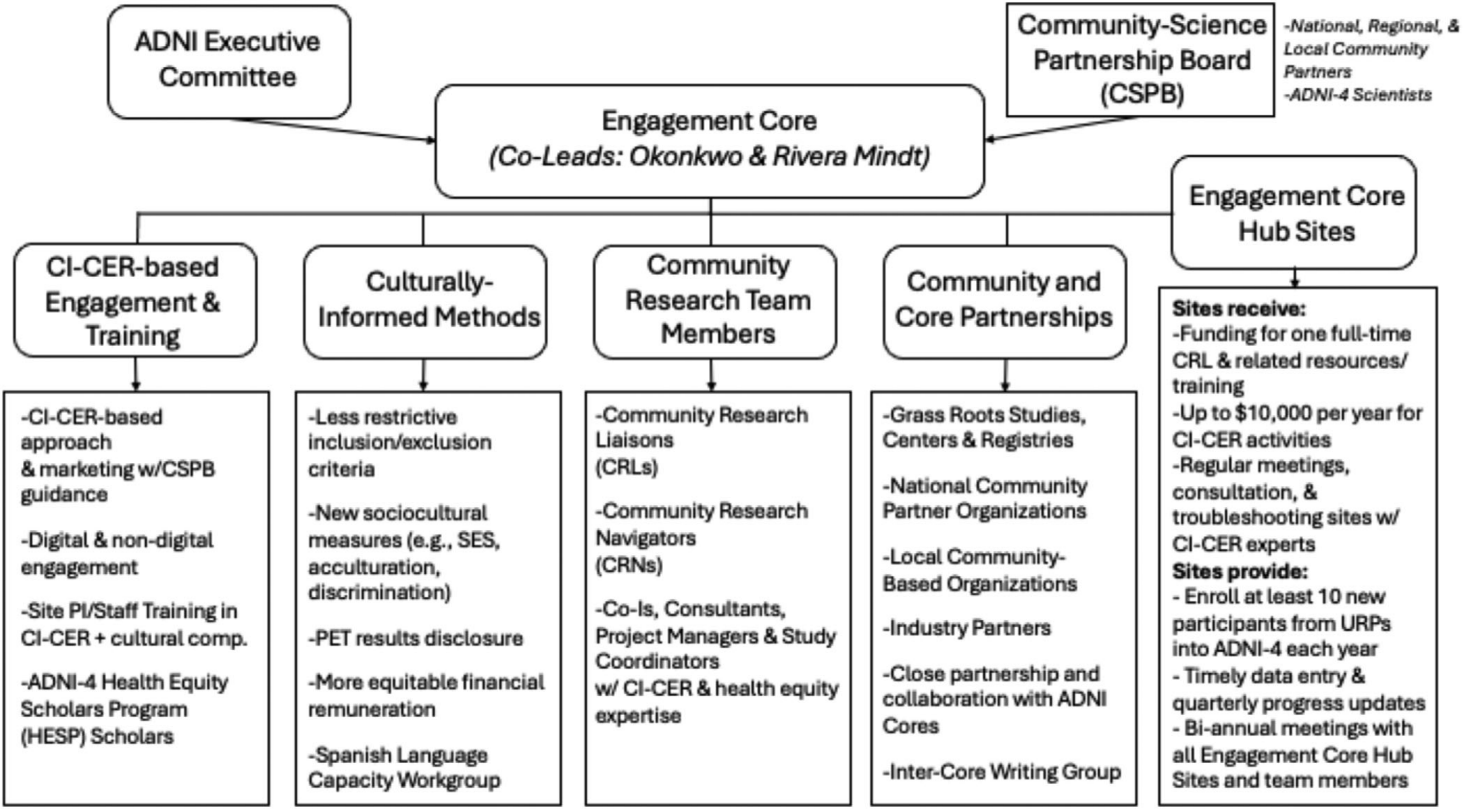
ADNI‐4 engagement core's organizational structure for implementation of the CI‐CER model. ADNI, Alzheimer's Disease Neuroimaging Initiative; CI‐CER, culturally informed, community‐engaged research; PET, positron emission tomography; SES, socioeconomic status; URP, underrepresented population.

The ADNI‐4 Engagement Core uses an intensive, CI‐CER approach. Figure 1 summarizes our scalable implementation of this evidence‐based model designed to reduce inequities in URP inclusion and engagement. Similar strategies have led to improved URP representation in AD research.^53^ Below, we highlight key components of this comprehensive, multifaceted model:

#### 2.2.1 Culturally informed methods

ADNI‐4 applies several methodological improvements to reduce burden, increase trust, and reduce barriers to inclusion and engagement. Briefly, changes to ADNI‐4 include:

1. Less restrictive inclusion/exclusion criteria related to comorbidities such as vascular burden (as described above in sections 2.1.1 and 2.1.2);
2. Less restrictive requirements for LP and study partner, the former of which is now optional (for part of the ADNI‐4 protocol), as described in section 2.1.1;
3. Measurement of sociocultural and structural determinants of health, including measurement of acculturation, socioeconomic status (SES), and other related measures (see Table 2);
4. Optional disclosure of amyloid PET results;
5. Greater emphasis on consistent, available support for transportation to and from study visits, which began during ADNI‐3;
6. Better, more equitable financial remuneration to both participants and study partners, which was not provided in prior ADNI phases (i.e., remuneration was previously provided solely to participants). Compensation for time and participation is now more aligned with CI‐CER principles and reduces barriers to participation, particularly for participants with low SES;^63^ and
7. The launch of the Spanish Language Capacity Workgroup (detailed below).

**Spanish Language Capacity Workgroup**: A key goal of the Engagement Core is to make ADNI‐4 culturally and linguistically inclusive. As a first step toward this goal, Engagement Core and ADNI‐4 leadership initiated Spanish language capacity development. In prior phases of ADNI, Spanish language efforts were implemented in an ad hoc, idiosyncratic manner and Spanish capacity existed on a site‐by‐site basis due to a non‐centralized institutional review board. Thus, the Engagement Core created a Spanish Language Capacity Workgroup (SLCW; co‐led by Drs. Guzman and Rivera Mindt; both bilingual [Spanish/English] neuropsychologists) and partnered with the Administrative and Clinical Cores to develop a standardized Spanish language protocol and process for all ADNI‐4 sites.

*Spanish Language Capacity Certification, Quality Assurance, and Consultation Processes*. To ensure that the ADNI‐4 Spanish language protocol is administered and interpreted in a culturally and linguistically competent manner, the SLCW has created a comprehensive Spanish language capacity certification process.

1. Eligibility requirements: A site is considered Spanish‐capable if they have a bilingual (English/Spanish) neuropsychologist on staff and/or have at least one bilingual (English/Spanish) psychometrist.
2. Application and review: Sites interested in becoming certified as a Spanish language ADNI‐4 site submit their electronic applications to the SLCW. The SLCW reviews each application based on standardized criteria (detailed below). Sites must be deemed eligible by the SLCW to evaluate new ADNI‐4 participants in Spanish.
3. Certification process: Approved sites undergo staff training and certification to ensure that each ADNI‐4 Spanish site is well equipped to conduct culturally and linguistically competent evaluations and quality assurance procedures with Spanish‐speaking participants. The workgroup also helps sites serve and engage Spanish‐speaking individuals more effectively and minimize barriers to participation. As part of this certification process, this workgroup has provided a SLCW guidelines protocol to confirm that each site possesses the necessary resources, staff proficiency, and commitment to conduct evaluations with Spanish‐speaking participants effectively. Our goal is for 15% of sites to become certified as Spanish language ADNI‐4 sites.
4. Decentralized bilingual neuropsychology support: An additional innovation to this program is that we offer certification for sites without a bilingual neuropsychologist on staff. This is important given the dearth of bilingual neuropsychologists in the AD/ADRD field. To address this AD/ADRD research capacity gap, the SLCW also established a protocol to guide and support sites without in‐person bilingual neuropsychologists. Specifically, the SLCW has developed a decentralized Spanish language training, supervision, and consultation program with our Core's staff bilingual neuropsychologist. Ongoing collaboration with the ADNI‐4 Engagement Core's bilingual neuropsychologist is required for sites *without* a bilingual neuropsychologist but *with* a bilingual psychometrist to ensure psychometrists’ training and proficiency, coordination of supervision for Spanish evaluations, and adherence to standardized assessment procedures.
5. Quality assurance and consultation service to ADNI‐4 Spanish sites: In addition to supervising the sites’ application and certification process, the SLCW also oversees certified Spanish sites to ensure ongoing compliance with established quality assurance standards and guidelines, and provides consultation and troubleshooting to sites for anything related to the Spanish language evaluations. For example, the ADNI‐4 Engagement Core's bilingual neuropsychologist (detailed above) meets remotely with site bilingual psychometrists to assess fluency, complete neuropsychological assessment training, certify psychometrists, and provide routine supervision. In addition, this bilingual neuropsychologist also collaborates with the site neuropsychologist/attending clinician to assist in the interpretation of neurocognitive results and provide any consultation support needed. These standards and processes ensure high‐quality Spanish language data for ADNI‐4.

#### 2.2.2 Inclusive participation and engagement methods

**Organizational Structure and Functions**: As detailed in Figure 1, the ADNI‐4 Engagement Core collaborates with all ADNI Cores to ensure that URPs have full and immersive participation in all study components and works closely with the Administrative and Clinical Cores given their highly interconnected inclusion and engagement goals and activities. The Engagement Core comprises an ethnoculturally and linguistically diverse team (detailed below). Highly experienced project managers (active = 2) and study coordinator (active = 1, target total = 2) provide direct supervision to this team. Regular contact (e.g., monthly Zoom conference calls for the entire team; monthly ADNI Executive Committee calls; national meetings, including the ADNI Steering Committee meeting; monthly Clinical Core meetings) is maintained across the Core, in addition to site‐specific meetings to discuss progress, challenges, and needs. Last, to advance the Engagement Core's Aim 3 (advance health equity research in ADNI‐4), the Core convenes a monthly “Inter‐Core Writing Group,” led by the Health Equity Scientists (active = 3), in which other Cores are invited to attend and regularly participate (e.g., Administration Core, Biostatistics Core) to collaborate on health‐equity and related papers and presentations.

The Engagement Core project managers oversee timelines and milestones for Engagement Core administrative, CI‐CER, and other scientific activities. To ensure inclusion and engagement goals are met for all study components, project managers work closely with the Engagement Core Hub Sites (detailed below) and community partners. They also work closely with the Engagement Core Co‐PIs, Lead Community Research Navigators (CRNs), Lead Community Research Liaison (CRL), and our Community‐Science Partnership Board (detailed below) to facilitate the development and implementation of CI‐CER–related outreach and educational materials for public‐facing collaborators. Additionally, Engagement Core project managers steward trainings and resource materials to promote CI‐CER and cultural competence of ADNI‐wide team members, including Site PIs and team members (e.g., resource repositories, in‐person and webinar trainings).

**Community Research Team Members**: A core feature of our CI‐CER approach is the depth and breadth of our community‐embedded team members, including CRLs and CRNs.

***CRLs (active = 3, total target = 1 per Hub Site)*** are outreach and inclusion specialists who promote URP inclusion through primarily in‐person, active engagement with local community‐based organizations (CBOs) by building trust through the Engagement Core's CI‐CER approach. They serve as a bridge between community members/CBOs and our ADNI‐4 in‐clinic sites, which are mostly located in large academic medical centers. CRLs are located within Engagement Core Hub Sites (detailed below) and work closely with the community, their local clinical site, and the Engagement Core for training, support, and guidance. At present, each Engagement Core Hub Site either has their own CRL on staff or is actively in the recruitment process for one (i.e., three active CRLs), and more will join our team as these efforts expand, for a target of one CRL per Hub Site.

Core responsibilities for CRLs include deploying local inclusion and engagement efforts, while bringing value to the community (the “give”). They are our “boots on the ground” who cultivate and maintain relationships with CBOs and community members. CRLs are involved in the referral process and conduct eligibility screenings. They attend community events at partner sites and present brain health education and workshops to CBOs. They directly link potential participants with whom they engage to their local ADNI site. CRLs are supervised by an experienced Lead CRL, who is in turn supervised by our Project Manager dedicated to the oversight of our CRL program. CRLs receive extensive training through a CRL‐specific orientation, a series of ADNI‐focused webinars, a detailed CRL standard operating procedures (SOP) manual, and a series of independent trainings on AD/ADRD, with topics such as brain health equity in older adults, inclusion techniques and awareness, community asset mapping, cultural competence and cultural humility, and using CI‐CER research approaches.

During the onboarding process, CRLs are trained on core responsibilities, including their scope of work, along with a toolkit and frequently asked questions for effective engagement; annual recruitment objectives and CI‐CER engagement approaches, such as community presentations, managing tables (i.e., “tabling”) at local events (e.g., at health fairs, community events), and e‐mailing with community partners. CRLs are also oriented to ADNI‐4 CI‐CER infrastructure including procedures for requesting community funding, educational materials, and ADNI‐4 branded items. They are educated about key questions frequently asked by community members. CRLs receive formal feedback on their performance and certification prior to engaging with the community, and complete detailed activity reporting and documentation trackers. Overall, the CRLs serve as ADNI‐4 community ambassadors and facilitate our ambitious inclusion goals by focusing on local referrals. With a warm handoff, eligible participants are referred from our CRLs to our clinical site staff.

***CRNs (active = 6, target total = 23)*** promote inclusion and engagement by providing *remote* navigational support to participants and study partners. These positions build on Care Navigator and “Promotora” models of navigational support^64^, ^65^, ^66^ to provide more intensive support to maximize engagement. Specifically, CRNs provide virtual assistance (e.g., phone, e‐mail, and/or online chat) that guides participants through every step of their ADNI‐4 journey, particularly the digital phase (see Miller et al., this issue)^61^ to help bridge the digital divide in which some participants may otherwise struggle. As a primary point of contact, CRNs help ease participant burden and cultivate trust. CRNs ensure that participants feel welcomed and are available to clarify complex study procedures, explain necessary preparation for visits (e.g., help with scheduling a Quest Diagnostics blood draw, explain standard MRI or PET procedures), and may provide links or resources to assist participants or study partners in related care.

CRNs work within the Engagement Core and are trained in effective communication, conflict management, and technology assistance, as well as cultural competence and cultural humility. Co‐learning is ongoing through monthly journal clubs and weekly training activities. CRNs serve a “help desk” function for our digital marketing efforts for potential and active participants who have study‐related questions, and these “help desk” functions are tracked through Zendesk, an online platform for remote participant contact via e‐mail, phone, or chat.

CRNs are supervised by one or more experienced Lead CRN, as well as a Project Manager dedicated to the oversight of our CRN program. Lead CRNs also respond to comments on ADNI social media, which aids in inclusion and engagement efforts. CRNs receive extensive training through a CRN‐specific orientation, a series of ADNI‐focused webinars, a detailed CRN SOP manual, and a series of independent trainings on AD/ADRD, with topics such as brain health equity in older adults, cultural competence and cultural humility, technical troubleshooting, ADNI knowledge, and using CI‐CER research approaches. Weekly meetings of Engagement Core Project Managers, Lead CRNs, and Administrative Core Project Managers ensure participant feedback is consistently reviewed and changes can be made in real time to improve participant experience, especially related to the remote cohort study activities. CRNs also help facilitate referrals from remote digital and/or blood cohorts to clinical site staff for future in‐clinic screening or when remote participants are not responsive to automated e‐mails. CRNs lend a personal touch to this process, which has increased successful referrals from digital and blood cohorts to in‐clinic sites.

The Lead CRN(s) and Project Manager provide CRNs provide formal feedback on their performance and final certification prior to engaging with potential or current participants or study partners. Overall, the CRNs serve as ADNI‐4 remote support and facilitate our retention and study task completion goals by helping participants and their study partners when they have questions or need study support.

**Engagement Core Hub Sites and Partnerships**: Engagement Core Hub Sites are select ADNI in‐clinic sites that receive additional financial and instrumental support for accelerated URP inclusion and engagement. Hub Sites are selected based on local demographics, the site's strong track record of or potential for URP engagement, presence of multiple local ADNI sites, and high capacity to enroll new participants. To date, the Engagement Core has established seven Hub Sites across the continental United States, including sites in the Northeast, Midwest, South, and West. Sites receive CI‐CER training and benefit from “lessons learned” from other hub and in‐clinic sites, which inform inclusion efforts throughout ADNI‐4. Hub Sites receive:

1. Funding for one full‐time CRL (as described above), as well as the opportunity to directly hire, train, and supervise the site's CRL in close coordination with the Engagement Core;
2. Up to $10,000 per year in additional funds to support partnerships with local CBOs, with the focus of establishing a presence and giving back to the community;
3. Regular meetings and as‐needed consultation for real‐time troubleshooting with Engagement Core team members;
4. Membership in a dynamic and engaged network of co‐learners, that is, fellow ADNI‐4 Engagement Core Hub Sites; and
5. A tailored, intensive digital advertising campaign to drive potential participants to their websites, if they choose (an option also available to other in‐clinic sites).

Engagement Core Hub Sites agree to the following:

1. Enrolling at least 10 new participants from URPs into ADNI‐4 each year;
2. Providing timely data entry;
3. Submitting quarterly status updates, describing progress, successes, and barriers encountered;
4. Participating in quarterly Engagement Core team meetings and bi‐annual meetings with all Engagement Core Hub Sites; and
5. Apprising the Engagement Core of any difficulties when support is needed.

**The ADNI CSPB (members = 17)**: provides guidance and iterative feedback on the inclusion and engagement of URP participants in ADNI. The board translates knowledge into actionable, culturally informed engagement strategies, prioritizing cultural humility and bidirectional communication. Motivators and barriers to research participation, including factors like mistrust, are considered. The CSPB plays a key role in comprehensive marketing efforts, including culturally informed participant communications. They are involved in planning and reviewing marketing and inclusion/engagement materials before implementation. Community members’ priorities are fully considered, feedback is typically integrated, and all decisions are transparently communicated. Overall, the CSPB is highly engaged throughout the course of the study, from implementation to dissemination.

Consistent with CI‐CER principles of equity and co‐learning,^56^ the ADNI‐4 CSPB is co‐chaired by four individuals: the two Engagement Core Co‐PIs (O. Okonkwo and M. Rivera Mindt) and two community members (B. Easter and S. Talavera), and has 13 additional standing members (the ADNI‐4 PI, M. Weiner; 6 community members; 6 scientific experts) with interest or expertise in dementia inequities and/or inclusion science, reflecting the diversity of communities served. In selecting community members, care was given to represent a variety of genders, socioeconomic levels, and geographic locations from across the United States. In addition, virtually all CSPB leadership and members self‐identify with an ethnoculturally minoritized URP group (i.e., American Indian/Alaska Native, Asian/Asian American, Black, and/or Latinx). Additional ADNI team members (e.g., leaders from other Cores, project managers) and representatives from ADNI‐4's marketing agency serve as ex officio members (≈ 25 individuals). The CSPB convenes quarterly for 2‐hour meetings with paid compensation for members’ time during these meetings and for any work (e.g., reviewing materials) completed between meetings.

The Engagement Core has also sought out guidance from national organizations (e.g., Alzheimer's Association), academic partners (e.g., Alzheimer's Disease Research Center Outreach Recruitment, and Engagement Core Committee members), local organizations (e.g., CBOs across our Engagement Core Hub Sites) and industry partners (e.g., ADNI Private Partner Scientific Board Diversity Work Group) to help support our URP inclusion and engagement efforts. These efforts include providing ADNI‐4 Ambassadors and referrals, working to facilitate digital and in‐person inclusion campaigns, referring representatives to serve on our CSPB, and offering opportunities to work directly with our ADNI HESP scholars to increase their knowledge of CI‐CER–based methods.

## 3 MEASURES

### 3.1 Sociocultural measures

As detailed above and in Table 3, for the first time ADNI has included a battery of sociocultural measures. The content areas of these measures include: acculturation, SES, quality of education, discrimination, stress, and rurality. Data from these measures will be available in future articles.

**TABLE 3:** Sociocultural determinants of health measures added to ADNI‐4.

Scales	Measures	Reference
Abbreviated Multidimensional Acculturation Scale (AMAS)	Acculturation	Zea et al., 2003^80^
Area Deprivation Index (ADI) score	Neighborhood socioeconomic disadvantage	Singh, 2019^81^
Hollingshead index score	Socioeconomic status	Hollingshead, 1957; Rentz & Raman, 2024^82^, ^83^
American National Adult Reading Test (AMNART)	Quality of education	Grober et al., 1991^84^
Brief Perceived Ethnic Discrimination Questionnaire (Brief PEDQ‐CV)	Discrimination	Contrada et al., 2001^85^
Perceived Stress Scale (PSS)	Stress	Cohen et al., 1983^86^
Rural–Urban Commuting Area (RUCA)	Rurality	USDA, 2022^62^
Rural–Urban Continuum Codes (RUCC)	Rurality	USDA, 2023^87^

### 3.2 Inclusion measures

Data used in the preparation of this article were obtained from the ADNI database (adni.loni.usc.edu). The ADNI was launched in 2003 as a public–private partnership, led by Principal Investigator Michael W. Weiner, MD. The primary goal of ADNI has been to test whether serial MRI, PET, other biological markers, and clinical and neuropsychological assessment can be combined to measure the progression of mild cognitive impairment (MCI) and early AD. The Alzheimer's Therapeutic Research Institute (ATRI)’s remote Electronic Data Capture (EDC) system is used to continually monitor the effectiveness of engagement strategies by tracking ADNI‐4 screening, inclusion, and engagement over time. The novel EDC system, which handles the complex clinical data management required by multisite projects such as ADNI, has been previously described in detail.^67^

Data reported here were current as of April 11, 2024, and reflect in‐clinic cohort data from 32 clinical sites, including 7 active Engagement Core Hub Sites. Rates and characteristics of new ADNI‐4 enrollment are reported. New enrollees are defined as participants enrolled in ADNI‐4 who have completed their baseline visit, and have not participated in previous phases of ADNI. Participants are considered to be from a URP background based on either ethnoculturally minoritized background, lower educational background, or rural geographic location, as detailed in section 2.1. Hub Sites were considered active as of the first date of participant enrollment. For additional information regarding sources/measures for other ADNI‐4 data (e.g., digital, blood cohorts), please refer to the other articles in this special issue (e.g., Miller et al. in this issue).^61^

ADNI‐4 data were compared to participant data from all prior ADNI phases, which was previously aggregated and used to determine URP representation; recent work highlights these inclusion inequities in previous ADNI cohorts (ADNI‐1 through ADNI‐3). Briefly, of the enrolled ADNI participants in previous phases (*N *= 2286), ≈ 87% were NLW, 5% were non‐Latinx Black (NLB), 4% were Latinx, 2% were non‐Latinx Asian/Asian American, and only 16% had ≤ 12 years of education. Compared to NLW and Asian/Asian American participants, NLB and Latinx participants were younger, more likely to be women, and had fewer years of education.^68^

## 4 RESULTS

In ADNI‐4, our goal is for individuals from URPs to represent at least 50% of newly enrolled participants at in‐clinic sites. Current, preliminary data demonstrate the following:

1. **ADNI‐4 new in‐clinic enrollment**: As of April 11, 2024, in‐clinic enrollment across 32 sites (including our 7 active Hub Sites) shows a total of 35 new enrollees, of which 21 are from URPs. Briefly, of the 35 new enrollees, 18 participants (51%) are from ethnoculturally minoritized backgrounds, including 3 who identified as multiracial (e.g., 2 identified as both NLB and NLW; 1 identified as American Indian or Alaskan Native and NLW). Five participants (14%) reported ≤ 12 years of education; of note, two enrollees reported both ethnoculturally minoritized backgrounds and ≤ 12 years of education. Overall, for new enrollees, 60% (21 out of 35) are from ethnoculturally minoritized or lower educational backgrounds. No new enrollees are yet from rural geographic locations.
  By diagnostic category, of the 35 new enrollees, 12 were cognitively unimpaired, 14 had MCI, and 9 met criteria for dementia. Of the 18 participants from ethnoculturally minoritized backgrounds, 8 were cognitively unimpaired, 7 had MCI, and 3 met criteria for dementia. Of the five participants who reported ≤ 12 years of education, one was cognitively unimpaired, three had MCI, and one met criteria for dementia.
2. **Hub Site enrollment**: Within our Engagement Core Hub Sites, specifically, our goal is to enroll ≈ 10 new URP participants each year. To date, seven Engagement Core Hub Sites are active, three of which have begun new participant enrollment. Although no Hub Sites have been active for a full year, one site has been active for 8 months and has already enrolled 11 new participants, 7 (64%) of whom are from a URP based on ethnoculturally minoritized or lower educational background. Cumulatively, across the three Hub Sites that have begun enrollment, 67% (14 out of 21) of all new enrollees are from URPs.
3. **Spanish assessment capacity**: Currently, 11 ADNI‐4 in‐clinic sites have applied for eligibility to conduct assessments with Spanish‐speaking participants. To date, five sites have been conditionally approved, contingent upon their continued collaboration with the Engagement Core's bilingual neuropsychologist, while three sites are still under review. Finally, three sites have been deemed ineligible due to the absence of either a bilingual (English/Spanish) staff neuropsychologist or psychometrist.

In addition, preliminary community‐level efforts, reflecting the work of our CRLs, CRNs, and CSPB to date in ADNI‐4 (i.e., from September 2022 to July 2024) are worth highlighting. Three CRLs have engaged in community outreach through various CBOs, such as local Alzheimer's Association chapters, academic medical centers and hospitals, senior housing sites, and faith‐based organizations by participating on boards or subcommittees (e.g., the East Harlem Community Health Committee), attending community events, and giving brain health and related community presentations to empower communities and strengthen relationships with community members. There have been 34 community education/engagement presentations, 19 tabling/health fair events, and 28 networking events which resulted in ≈ 57 referrals for new ADNI‐4 enrollment. Our six active CRNs have supported participants through the digital platform and handle ongoing social media responses to marketing material (e.g., Facebook advertisements; see Nosheny et al.^69^ The ADNI Administrative Core: Ensuring ADNI's success and informing future AD clinical trials). The ADNI CSPB has met quarterly since its inception in 2022, and has been an integral part of ADNI‐4's accomplishments and impact. Herein we provide some highlights of the CSPB's substantial contributions. First, the CSPB informed and supported the development of the Engagement Core's component of the ADNI‐4 grant application, which resulted in the successful funding of this new core to ADNI. Second, the CSPB successfully advocated for and provided iterative feedback on the materials and protocol for ADNI‐4's newly launched amyloid PET results disclosure for participants. Third, the CSPB helped guide the development, refinement, and implementation of ADNI's new Sociocultural and Structural Determinants of Health Battery (please see Table 2). Fourth, the CSPB has made significant contributions to the development and ongoing refinement of ADNI‐4's inclusion/engagement materials and strategies. For example, the CSPB reviews and provides feedback on all marketing materials used in ADNI‐4 prior to dissemination and reviewed and provided input on ADNI‐4's consent documents. They receive ongoing updates and provide input on participant enrollment, including Hub Site approvals, and the HESP. Fifth, CSPB members have co‐authored two ADNI‐4 publications (i.e., this article and Amaza et al., in this issue).

In addition to increasing the diversity of the ADNI‐4 cohort (as reported above), the Engagement Core also seeks to advance health equity research by increasing AD/ADRD scientific workforce capacity through CI‐CER and cultural competence training for all ADNI leadership, team members, and our HESP scholars. In brief, HESP provides funding and mentorship for diverse and/or equity‐focused early career scholars and is described further in Amaza et al., in this issue. Finally, in terms of scholarly contributions to advance brain health equity in AD/ADRD research, ADNI‐4, led by the Engagement Core, has disseminated this work (e.g., methods of ADNI‐4, preliminary results for ADNI‐4, and/or publications/presentations produced through our ADNI‐4 writing group) through four peer‐reviewed publications^31^, ^70^, ^71^, ^72^ and seven presentations.^73^, ^74^, ^75^, ^76^, ^77^, ^78^, ^79^

## 5 DISCUSSION

### 5.1 Overview and implications

Overall, as of April 2024, 60% of ADNI‐4 new enrollees are participants from URPs based on ethnoculturally minoritized or lower educational background. Therefore, our CI‐CER efforts to date already exceed our ≥ 50% URP inclusion goal. This level of URP inclusion early in ADNI‐4 is particularly noteworthy given that the combined past ADNI cohorts (ADNI‐1, ADNI‐GO, ADNI‐2, and ADNI‐3) only reached ≈ 20% URP inclusion.^20^, ^68^

For 20 years, ADNI has been one of the largest and most impactful AD studies in the field. At the same time, like the broader field of AD/ADRD research, there have been significant limitations in the external and internal validity of ADNI due to its historical under‐inclusion of persons from URPs (i.e., ethnoculturally minoritized background, low education, rural geographic location) and lack of culturally informed methods to assess and characterize AD/ADRD‐related brain–behavior relationships in URPs. These scientific limitations take on even greater salience as the intersectional implications of an increasingly older and more ethnoculturally diverse population continue to emerge in the United States in the coming decades.

ADNI‐3 launched an ambitious pilot project, the ADNI‐3 DVTF, to examine the feasibility and scalability of implementing a comprehensive CI‐CER model at a subset of ADNI‐3 sites to address these scientific challenges. The successful results of that pilot prompted the addition of the Engagement Core to ADNI‐4. Unlike the rest of the ADNI‐4 Cores, the Engagement Core is entirely new and has required substantial investments of time, resources, expertise, and person power to build the necessary infrastructure to launch an evidence‐based, CI‐CER model of such size and scope. In close collaboration with the other ADNI‐4 Cores, this Core aims to enact significant and sustained increases in the inclusion and engagement of persons from URPs by applying CI‐CER methodology scaled for broad, multisite implementation; cultivate a culturally competent workforce within ADNI‐4 and in the AD/ADRD field more broadly (see Amaza et al. in this special issue for further details); and advance health equity science within ADNI‐4. Preliminary data show that ADNI‐4 is already exceeding its inclusion goals of at least 50% URP inclusion, with 60% of new in‐clinic enrollees and 67% of new Hub Site enrollees from URPs based on ethnoculturally minoritized or lower educational background. If this level of URP inclusion and engagement continues, this will significantly improve the external validity of ADNI results to the US population. ADNI‐4 could serve as a model for scaling CI‐CER approaches in both a meaningful and sustainable manner, which could be deployed in other large‐scale observational studies and clinical trials.

### 5.2 Limitations and strengths

It is imperative to highlight the limitations of this work. First, the intrinsic epistemological and ontological shifts involved in evolving such a large, ongoing study as ADNI from a traditional clinical research model to a CI‐CER model will inevitably slow data collection, at least initially, and involve some growing pains along the way. Second, the metrics used in this study need to be refined further for deeper characterization of these efforts and greater impact from an inclusion science perspective. Third, increasing inclusion and engagement rates in specific populations (e.g., some American Indian/Alaska Native, Asian/Asian American older adults) will require significant investment and resources for culturally informed materials and engagement efforts (e.g., language translations, outreach to new community groups). Fourth and finally, while these CI‐CER methods and preliminary findings are promising, ADNI‐4 is still in its nascence. It remains to be seen if this high level of URP inclusion will be sustained at a national level as ADNI‐4 continues.

Despite these limitations, the Engagement Core also has important strengths that merit discussion. First, this is an ambitious, novel, and significant effort based on robust prior empirical evidence.^31^ Second, if a study as large and well established as ADNI can make these shifts to better meet the demographic and scientific challenges of the moment, this work can inform AD clinical trials to do the same, which is urgently needed and long overdue. Third, advancing the scientific validity of ADNI helps provide a roadmap for the AD/ADRD field as a whole to become more relevant, ethical, and just.

### 5.3 Future directions

As we continue to collect data, we will evaluate the effectiveness of our CI‐CER approach by monitoring inclusion and engagement rates among URPs in ADNI‐4 over time creating more nuanced metrics of community engagement. Specifically, we will examine: (1) rates of completed baseline visits; (2) rates of sustained engagement defined as completion of follow‐up visits (retention); (3) successful completion of each component of the ADNI study (e.g., participation in remote cohort activities, in‐clinic visits, MRI, PET, brain donation); (4) the efficacy of different types of CI‐CER efforts (e.g., in‐person/CRL vs. digital); and (5) rates of exclusion due to comorbidities and other factors across underrepresented participants, including ethnocultural, rural, and educational attainment groups. We will compare these rates across URP groups, to our current inclusion and engagement goals, and to previous ADNI cohorts. Ultimately, we will examine the association between these metrics and demographic and sociocultural characteristics that may be differentially associated with inclusion and engagement outcomes in URPs.

In sum, led by the Engagement Core and in close partnership with all ADNI‐4 Cores, we will continue to implement and refine these CI‐CER methods across our sites, significantly increasing the inclusion of URP participants and thus improving the generalizability of future ADNI data to the US population, particularly for those who have been historically excluded from research. This approach is essential for the overarching success of ADNI and has important implications for advancing inclusion science, informing clinical trials, and addressing the urgent shortage of culturally competent researchers in the AD/ADRD workforce.

## CONFLICT OF INTEREST STATEMENT

Please see submitted author ICJME disclosure forms. Author disclosures are available in the supporting information.

## CONSENT STATEMENT

All human subjects provided informed consent.

## Supporting information

Supporting Information
